# The Neurotoxicity of DOPAL: Behavioral and Stereological Evidence for Its Role in Parkinson Disease Pathogenesis

**DOI:** 10.1371/journal.pone.0015251

**Published:** 2010-12-13

**Authors:** W. Michael Panneton, V. B. Kumar, Qi Gan, William J. Burke, James E. Galvin

**Affiliations:** 1 Department of Pharmacological and Physiological Science, Saint Louis University School of Medicine, St. Louis, Missouri, United States of America; 2 Department of Geriatrics, Saint Louis University School of Medicine, St Louis, Missouri, United States of America; 3 Department of Neurology, Saint Louis University School of Medicine, St. Louis, Missouri, United States of America; 4 Department of Neurology and Department of Psychiatry, Center of Excellence on Brain Aging, New York University Langone Medical Center, New York, New York, United States of America; Nathan Kline Institute/New York University, United States of America

## Abstract

**Background:**

The etiology of Parkinson disease (PD) has yet to be fully elucidated. We examined the consequences of injections of 3,4-dihydroxyphenylacetaldehyde (DOPAL), a toxic metabolite of dopamine, into the substantia nigra of rats on motor behavior and neuronal survival.

**Methods/Principal Findings:**

A total of 800 nl/rat of DOPAL (1 µg/200 nl) was injected stereotaxically into the substantia nigra over three sites while control animals received similar injections of phosphate buffered saline. Rotational behavior of these rats was analyzed, optical density of striatal tyrosine hydroxylase was calculated, and unbiased stereological counts of the substantia nigra were made. The rats showed significant rotational asymmetry ipsilateral to the lesion, supporting disruption of dopaminergic nigrostriatal projections. Such disruption was verified since the density of striatal tyrosine hydroxylase decreased significantly (p<0.001) on the side ipsilateral to the DOPAL injections when compared to the non-injected side. Stereological counts of neurons stained for Nissl in *pars compacta* of the substantia nigra significantly decreased (p<0.001) from control values, while counts of those in *pars reticulata* were unchanged after DOPAL injections. Counts of neurons immunostained for tyrosine hydroxylase also showed a significant (p = 0.032) loss of dopaminergic neurons. In spite of significant loss of dopaminergic neurons, DOPAL injections did not induce significant glial reaction in the substantia nigra.

**Conclusions:**

The present study provides the first *in vivo* quantification of substantia nigra *pars compacta* neuronal loss after injection of the endogenous toxin DOPAL. The results demonstrate that injections of DOPAL selectively kills SN DA neurons, suggests loss of striatal DA terminals, spares non-dopaminergic neurons of the *pars reticulata*, and triggers a behavioral phenotype (rotational asymmetry) consistent with other PD animal models. This study supports the “catecholaldehyde hypothesis” as an important link for the etiology of sporadic PD.

## Introduction

Parkinson disease (PD) is the most common neurodegenerative movement disorder, affecting 2% of individuals over age 65 and 4–5% over 85 years [1]. PD is characterized phenotypically by bradykinesia, tremor at rest, rigidity and postural rigidity, and pathologically by the loss of dopaminergic neurons in the substantia nigra (SN), severe dopamine (DA) loss in the striatum and the accumulation of alpha-synuclein (α-syn). Although the exact causes of PD remain unknown, it is likely a combination of several factors.

Many interrelated hypotheses have been postulated about the death of dopaminergic neurons including genetic defects [2], [3], environmental toxins [4]–[6], inflammation [7]–[9], deficiencies in the mitochondrial respiratory chain [10], [11], and reduced capacity of transmitters, including monoamine storage vesicles [12], [13] and glutamate metabolism [14], [15]. However, no experimental animal models testing these hypotheses show all the features characterizing PD. Moreover, most animal models of PD use exogenous toxins to kill dopaminergic neurons in the SN, which may not relate to cases of idiopathic PD in humans.

The involvement of DA or one of its metabolites also may be important in the death of DA SN neurons [16], [17]. The “catecholaldehyde hypothesis” of PD proposes that an accumulation of a toxic intermediate of dopamine metabolism, 3,4-dihydroxyphenylacetaldehyde (DOPAL), is toxic to nigral neurons and leads to PD. DOPAL is the catabolic product of dopamine via oxidative deamination by monoamine oxidase (MAO), and is quickly cleaved by aldehyde dehydrogenase (ALDH1A1) into 3,4-dihydroxyphenylacetic acid (DOPAC). DOPAL is an endogenous toxin found in dopaminergic cells in human SN [18], [19] and could contribute to the development of PD. Here we examine whether DOPAL selectively kills dopaminergic neurons in the SN.

Our laboratories have shown that DA itself is not sufficiently toxic at physiological levels to induce either neuronal death [19], [20] or aggregation of α-synuclein [21], thus implicating a metabolite of DA. Investigations in several laboratories have implicated a metabolite of DA as an endogenous toxin which triggers DA neuron loss [19], [20], [22]–[29]. DOPAL levels of 2–3 µM are normally present in SN from neurologically intact human patients at autopsy [19]. However, DOPAL levels increase in the SN and striatum in PD [30] while ALDH1A1 mRNA, protein and activity decrease in the SN and striatum [31], [32], [32]–[34], implicating DOPAL as a potential endogenous toxin. Moreover, we have shown that DOPAL is toxic to neurons at physiological concentrations *in vitro* [19], [21] and also triggers aggregation of α-synuclein [21]. Earlier experiments provided immunohistochemical evidence of DOPAL toxicity *in vivo* by showing loss of tyrosine hydroxylase immunoreactivity (THir) after DOPAL injections into rat SN [20], [21]. However these studies did not exclude the possibility that DOPAL injections may have decreased tyrosine hydroxylase (TH) synthesis and protein levels resulting in decreased THir as was shown for DA [35]. Here we determined that DOPAL induces loss of striatal DA *in vivo* using tyrosine hydroxylase immunohistochemistry and show that DOPAL is toxic to DA neurons *in vivo* with definitive neuronal counts using unbiased stereology [36]. In addition we show that DOPAL injections into SN produce a behavioral model of PD. The experiments provided herein strongly reinforce the notion that DOPAL is an endogenous neurotoxin, and implicate it as the trigger which kills dopaminergic neurons in the SN and leads to Parkinson disease.

## Results

### Behavioral Evaluation

**Figure 1:**
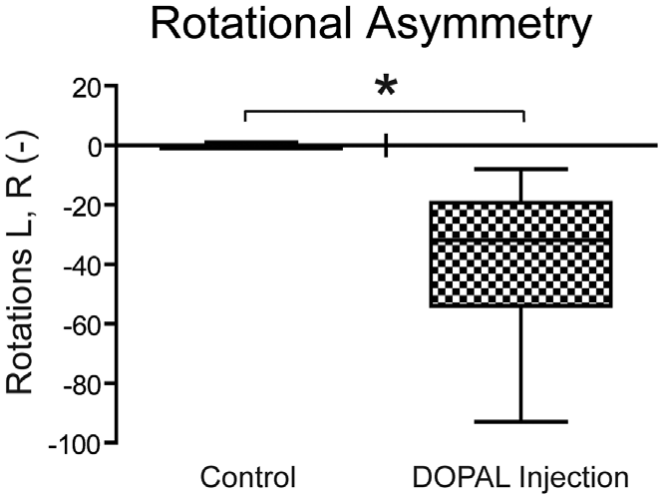
Box plot illustrating the behavioral changes in rats after unilateral injections of DOPAL into their substantia nigra. Rats showed rotational asymmetry, turning significantly towards the side of DOPAL injections. *p<0.05.

Rotational asymmetry was assessed to quantify the effect of unilateral depletions of striatal dopamine from disruptions of nigrostriatal circuitry. We show that rats significantly (p<0.05) prefer rotating to the side ipsilateral to the unilateral DOPAL injections versus control rats (Fig. 1) after injections of apomorphine.

### Neuropathological Evaluation: Immunohistochemistry

**Figure 2:**
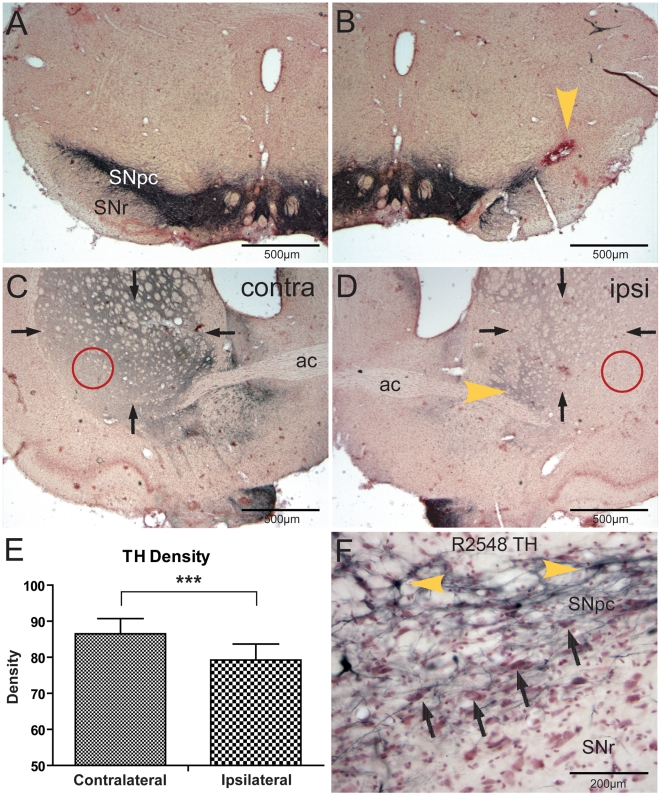
Photomicrographs of brain sections (case R2508) immunohistochemically-stained against tyrosine hydroxylase (TH) after injections of DOPAL into the substantia nigra, *pars compacta* (SNpc). Note the gross reduction of TH immunoreactivity in the SN at the site of injection (**B**; yellow arrowhead) versus the non-injected side (**A**). Similar loss of TH staining is seen in the striatum ipsilateral to the injection (**D**, arrows) versus that on the non-injected side (**C**, arrows), suggesting disruption of nigral dopaminergic terminals. The area just lateral to the anterior commissure (**D**, yellow arrowhead) however was always densely labeled (see text). Densitometry of immunostaining of striatal TH (**E**) showed significant differences (p<0.001) of the whole striatum contralateral and ipsilateral to DOPAL injections. Spot density measurements of ventrolateral parts of the striatum (**D**, red circles), however, showed an 80% loss of immunoreactivity ipsilateral to the injection. Intensely stained neurons with antibodies against tyrosine hydroxylase (**F**, yellow arrowheads) were sometimes seen in the SNpc of control brains surrounded by numerous neurons stained only for Nissl (**F**, black arrows), suggesting that counting only TH-immunostained neurons may be problematic. Abbreviations: ac, anterior commissure; SNpc, pars compacta of substantia nigra; SNpr, pars reticulata of substantia nigra. *** p<0.001.

In all cases there was a decrease in immunoreactivity of TH in the SN ipsilateral to the injections of DOPAL (Fig. 2B, yellow arrowhead) compared to the contralateral, non-injected side (Fig. 2A). There also was significantly (p<0.001) less TH immunoreactivity in the striatum on the side ipsilateral to the DOPAL injections (Fig. 2D, arrows; Fig. 2E) compared to the non-injected contralateral side (Fig. 2C, arrows; Fig. 2E). After background densities were subtracted, we calculated a 28% reduction in immunoreactivity in the striatum on the side ipsilateral to the DOPAL injections, suggesting a loss of DA terminals on the injected side. We noted that the ventrolateral striatum through levels of the globus pallidus were especially denervated (Fig. 2D, red circles). Spot density measurements contralateral (17.8±4.5 units) versus ipsilateral to the DOPAL injections (3.5±5.9 units) here were reduced 80%.

### Neuropathological Evaluation: Stereology

The SN was included in 8–10 sections of all cases counted, and its total length was approximately 1.25 mm. Mean volume of the SNpc of control rats was 268,639,250 µ^3^, while that of the SNpr was 777,696,500 µ^3^. Mean volume of the SNpc in the DOPAL-injected rats was 264,674,833 µ^3^ while that of the SNpr was 760,212,500 µ^3^. There was no significant difference in mean volumes of SNpc or SNpr between controls and DOPAL injected rats.

**Figure 3:**
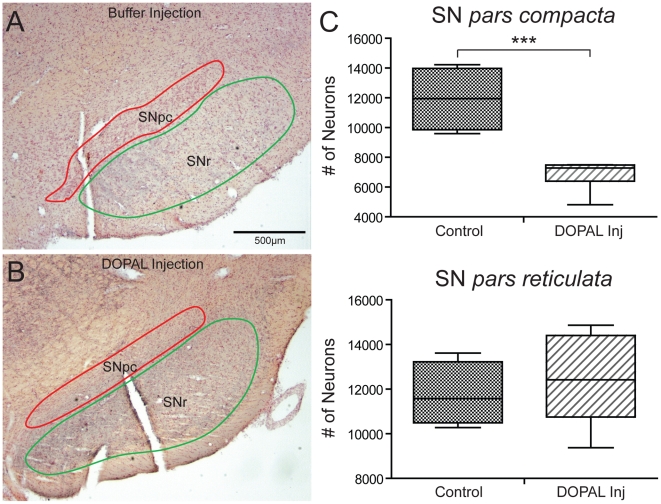
Photomicrographs of sections through the SN stained for Nissl with neutral red. Red lines mark the boundaries enclosing the substantia nigra, *pars compacta*, while green lines encompass the substantia nigra, *pars reticulata*. Unbiased stereological counts using optical fractionator probes were made of neurons in both SNpc and SNpr in sections from animals injected with buffer (**A;** case R2546) and those injected with DOPAL (**B,** case R2505). Note the significant (p≤0.001) loss of SNpc neurons in rats (**C**) after the DOPAL injection when compared to control rats, while no loss of neurons was seen in the adjacent SNpr.

We first counted TH immunoreactive neurons in the SNpc on the side of the DOPAL injection and compared them to those on the non-injected side. When only TH immunoreactive neurons were counted, the mean number of TH immunoreactive neurons ipsilateral to the DOPAL injections side was 50% less than that of the contralateral non-injected side, significantly different (p = 0.032) using the paired samples T-test by difference method (Table 1). However, we noted that numerous SNpc neurons sometimes were not stained for TH despite robust labeling of others (Fig. 2F). Thus, we compared the number of Nissl stained profiles in sections immunostained with α-syn rather than TH in the SNpc's ipsilateral to the DOPAL injections to those of control rats which had received injections of a buffered saline solution into their SN's (Table 1). The number of Nissl-stained neurons in the SNpc (compare Figs. 3A, B) of the DOPAL injected rats was 43% less than that of the saline-injected rats (Fig. 3C) which was significantly different (p≤0.001). We then determined whether DOPAL was toxic to neurons in the subjacent *pars reticulata* of the SN. The number of neurons in the SNpr of the DOPAL-injected rats was not different from the saline-injected rats (Table 1; Fig. 3C). This suggests that DOPAL is selectively lethal to dopaminergic neurons in the SNpc, further supporting the catecholaldehyde hypothesis.

**Table 1:** Toxic Effect of DOPAL on Substantia Nigra Neurons.

	TOTAL SNpc Neurons	TOTAL SNr Neurons	TH SNpc Neurons
**Control**	11926 (1084)	12422 (832)	11969 (3699)
**Experimental**	6879 (422)	11761 (715)	5884 (1365)
**p-value**	0.001	n.s.	0.032

Means (SD); n.s.  =  not significant.

The effect of DOPAL injections into the substantia nigra on neurons in either the *pars compacta* (SNpc) or the *pars reticularis* (SNpr) are shown. Control animals were injected with buffered saline while experimental animals were injected with DOPAL (4 µg/800 nl). Unbiased stereology was used to assess the number of neurons (see Methods).

### Neuropathological Evaluation: Activation of Glia

**Figure 4:**
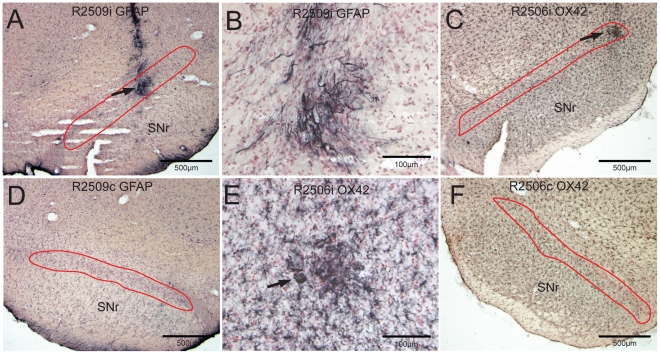
Brightfield photomicrographs of sections through rat brains stained immunohistochemically for glia. Astrocytes were immunolabeled with glial fibrillary acidic protein (GFAP) in rats but only a few were found in the SN surrounding injections of DOPAL (**A**; arrow). A higher magnification of the injection site shown in **A** is seen in **B**. Numerous microglia stained immunohistochemically with antibodies against OX42 were found throughout the brain, but we considered few reactive (**C**, arrow). Some reactive microglia [see the large multinucleated phagocytic-like cells (**E;** arrow)] were close to injections, but these were not abundant in the SNpc. Compare the number of Nissl-stained neurons in the SNpc (**A, C, D,** and **F**, red outlines) on the side ipsilateral to the DOPAL injection (**A**, **C**) to those on the contralateral side (**D**, **F**); counts using unbiased stereology indicate 43% fewer neurons on the side of the injection but no loss in the juxtaposed SNpr.

We immunostained a series of sections against antibodies to glial fibrillary protein (GFAP), a marker for astrocytes, and to OX-42, which stains microglia, to determine the relationship between neuronal loss following DOPAL injections and the activation of glial cells. Reactive astrocytes were defined as cells with up-regulation of GFAP having pronounced hypertrophy of cell body and processes with considerable extension of these processes beyond the normal domains of individual astrocytes [37]. Reactive astrocytes and their processes were localized to and surrounded the injection site in some of our cases (Figs. 4A, B). There were no reactive processes streaming throughout the SNpc or SNpr though, suggesting that the major losses of neurons in the SNpc [compare neuronal density of the SNpc ipsilateral to injections (Figs. 3B, 4A, 4C) to that contralateral (Figs. 3A, 4D, 4F)] was not due to astrogliosis. Mild astrogliosis was noted in the ventromedial parts of the SNpr both ipsilateral and contralateral to DOPAL injections.

Activated microglia also were relatively few in absolute number after DOPAL injections (Fig. 4C, arrow) with only few activated cells (Fig. 4E, arrow). We counted 60 engorged microglia immunoreactive to OX-42 in the ipsilateral SNpc of the rats receiving DOPAL injections (n = 6), and 15 in the adjacent SNpr. However, most activated cells were detected in three DOPAL cases, with few or no activated microglia in other injected or control cases.

## Discussion

Our results show that DOPAL induces a behavioral phenotype (asymmetrical rotation), significantly decreased TH-immunoreactivity of nigrostriatal projections, and is lethal to neurons in the *pars compacta* of the SN but not those in the adjacent *pars reticulata* in rats. This study is the first to quantify *in vivo* the death of neurons in the SN due to an endogenous toxin naturally produced in dopaminergic neurons. It augments our *in vitro* data implicating DOPAL as a toxin and supports our contention that intracellular accumulations of DOPAL trigger death in dopaminergic neurons in the SNpc and may be relevant to the pathogenesis of PD in humans [19]–[21], [38].

Measurable levels of DOPAL are found in post-mortem human brains [18] and increased amounts of DOPAL are in autopsy brains of PD [30], [32]. We have shown previously that DOPAL is toxic to PC12 cells *in vitro* at dosages as low as 6.6 µM [19] and triggers aggregation of α-synuclein *in vitro* at dosages as low as 1.5 µM [21]. Previous studies with intracerebral injections of DOPAL used doses ranging between 0.05–0.75 µg/200 nl, with doses above 0.1 µg/200 nl destroying at least some of the DA neurons in the substantia nigra/ventral tegmental area of the midbrain [20], [21]. However, these studies did not exclude that loss of THir after DOPAL injections into SN was due to decreased TH synthesis [35]. In the present study we made three injections of 200 nl-400 nl-200 nl along the rostrocaudal extent of the SN, hoping to include all the DA neurons in the presumed ellipsoid shape of the injection. The DOPAL injections (1 µg/200 nl) caused neuronal loss only in the SNpc, while sparing neurons in the juxtaposed SNpr. This is of interest since between 71–80% of neurons in SNpc are dopaminergic [39], [40] while those in SNpr are approximately 70–80% GABAergic [39], suggesting that DOPAL may be selectively lethal to DA neurons.

We elected unilateral injections since bilateral disruption often results in aphagia, adipsia and high mortality rates [41], [42]. Most studies on rats inducing hemi-Parkinson symptoms use rather large (i.e., ≥4 µl) injections of 6-OHDA into their median forebrain bundles [43]–[45]. Numerous dopamine neurons in the ipsilateral SNpc are killed after such injections, resulting in loss of dopamine in the ipsilateral striatum but also in the prefrontal cortex, nucleus accumbens, septum and olfactory tubercles. Injecting DOPAL unilaterally into the SN also resulted in the ipsilateral loss of striatal TH immunoreactivity, and perhaps DA in nerve terminals of the striatum. Unilateral depletion of striatal DA also allows for tests comparing the dopamine innervation on either side of the striatum [43], [44], [46].

Tests such as rotational asymmetry determine imbalances in dopaminergic innervation and are easily evaluated [47]. Rotational behavior after unilateral nigral lesions is hypothesized to be dependent on the balance between striatal dopamine release and hypersensitivity of striatal dopamine receptors on the two sides [43]. We evaluated rotational asymmetry in rats injected with the endogenous toxin DOPAL into their SN, and show significant asymmetry with rats turning ipsilateral to the injection. Our rats turned to the same side as the lesion after apomorphine injection, similar to other studies after intranigral injections of toxins [43]. This finding is consistent with the typical asymmetrical onset of PD in humans.

Rotational asymmetry also is dependent on which neurons of the basal ganglia circuitry are involved. For example, turning behavior can be manipulated with lesions/stimulation of the prefrontal cortex [48], the centromedian-parafasicular-thalamic complex [49], the subthalamic nucleus [50], [51] or the SNpr [52], [53]. Indeed, killing neurons in both SNpc and SNpr induce different behaviors in rats than killing neurons in SNpc alone [52], [54]. Although our experiments targeted SNpc neurons, quantitative measurements show that SNpr neurons were spared. Thus the DOPAL model recapitulates many features of nigral degeneration in sporadic PD. We cannot however fully discount potential toxicity to non-dopaminergic neurons.

The importance of correct counts of neurons in the SN in the various animal models of PD has been emphasized [55], especially if comparisons of degeneration of nigral dopaminergic neurons among different studies are to be accurate. The volumes of either the SNpc or SNpr nuclei were similar in both our control and DOPAL-injected animals, suggesting uniformity in our interpretation of the nuclear outlines. Since shrinkage of tissue can be problematic when comparing counts from different processing methods, all tissues were processed similarly using free-floating sections. Moreover, since neurons in both the SNpc and SNpr subnuclei are neither homogeneous in size or distribution, the whole rostrocaudal extent of these subnuclei was included in analysis. The estimated number of neurons determined by these methods in the SNpc of our control rats (11926±1084) compares favorably with those using similar methodologies in both rats [39], [56] and mice (reviewed by Baquet et al., 2009), with numerous studies indicating between 8,000–12,000 TH-positive neurons in the SN.

We used Nissl stained sections for neuronal cell counts in the SN since there was large variability in counts of TH immunostained neurons, similar to observations of others [57], [58]. Our densitometry measurements of the whole striatum were also variable between cases, but collectively showed a 28% decrease on the side ipsilateral to the DOPAL injections. This number reflects the total TH immunostaining of the striatum, however, which includes catecholaminergic innervation from numerous sources such as the SNpc, the ventral tegmental and retrorubral areas, as well as the locus coeruleus [59]. For example, an area just lateral to the anterior commissure (Fig. 2D, yellow arrowhead), always was densely labeled ipsilateral to the DOPAL injections, and this area receives projections from a subdivision of the ventral tegmental nucleus [60]. The ventrolateral portion of the striatum through levels of the globus pallidus was especially devoid of immunoreactivity after DOPAL injections; spot density of this portion showed an 80% reduction over the control side. Nevertheless, studies have shown that neurons utilizing monoamines as transmitters/messengers vary their metabolism throughout the day [61], [62] and are asymmetrically lateralized [63]; this is especially true for neurons utilizing dopamine. These results should provide caution to those quantifying TH immunopositive neurons as their sole data to determine the extent of lesions of SN neurons.

Although reactive astrocytes and microglia have been implicated in the etiology of Parkinson disease [7], [8], [37], [64], we saw few reactive astrocytes or activated microglia in DOPAL-injected animals. Inflammatory cells are proposed to induce or mediate death to dopamine neurons in the SNpc [7]–[9], [64], however our results do not support a primary role of glial activation in SNpc degeneration, but instead may be a later event in the pathogenesis of PD.

The present study provides the first *in vivo* quantification of neuronal loss after injection of an endogenous toxin. The results demonstrate that injections of DOPAL kill SN DA neurons with loss of striatal DA terminals and also induce rotational asymmetry in rats. These results add to an increasing body of evidence obtained in our laboratories that the endogenous metabolite of dopamine, DOPAL, is toxic to dopaminergic neurons. We already have provided evidence that DOPAL, but not other dopamine metabolites, induces cell death *in vitro* [65] and *in vivo* [20], induces aggregation of α-synuclein [21], and disrupts mitochondrial function and creates reactive oxygen species [19], [25], [65]. DOPAL, like its analogue 3,4-dihydroxyphenylglycolaldehyde, also activates the mitochondrial permeability pore which can lead to apoptotic neuronal death [19], [65]–[67]. Moreover, this data is supported by work from others showing that DOPAL is increased in both the SN and striatum in PD brains [30], [32]. Here we provide definitive evidence that DOPAL is toxic *in vivo*, triggering a behavioral phenotype consistent with other PD animal models. These data thus support the catecholaldehyde hypothesis on the etiology of Parkinson disease.

## Materials and Methods

Ten adult male Sprague Dawley rats (275–299 g) were purchased commercially (Harlan Laboratories, Indianapolis, IN) and a housed in the Department of Comparative Medicine at Saint Louis University. All protocols were approved by the Animal Care Committee of Saint Louis University and followed the guidelines of the National Institutes of Health Guide for Care and Handling of Laboratory Animals.

### Behavioral Evaluation

A commonly used measurement of unilateral dopaminergic denervation of the rodent striatum is rotational asymmetry [47], [68] [69]–[71]. Rats were introduced to the test one week prior to surgery to establish baseline control data. Rats were injected subcutaneously with the dopamine agonist apomorphine (0.4 mg/kg) dissolved in 0.1% ascorbate saline solution [72]. After waiting 5 min, the rats were placed in a hemispheric rotation bowl 40 cm wide and 20 cm deep and the number of complete turns to the right or the left quantified by observation. This test was performed both prior to DOPAL injection and again one day prior to sacrifice.

### Surgery

The rats were anesthetized with injections (IP; 0.1 ml/kg) of a cocktail of ketamine (60 mg/ml) and xylazine (40 mg/ml) and mounted in a stereotaxic frame. DOPAL was synthesized as previously described [73] and dissolved immediately prior to injection in 1% benzyl alcohol then diluted to the final concentration (1 µg/200 nl) with phosphate buffered saline (PBS; pH 7.4) and red Fluorospheres (Molecular Probes, Eugene, OR). Three injections (200–400 nl-200 nl) of DOPAL were made through the rostral-caudal extent of the SN of six rats, using a glass micropipette (tip diameter 20–30 µm) attached to a 1 µl Hamilton syringe at coordinates AP +3.0, 3.6, 4.2; ML 2.0, 2.2, 2.1; DV+2.3, 2.2, 2.0. Control rats (n = 4) were injected similarly with the same volume of PBS into their SN. The micropipette remained in place for 5 min to help prevent spread of the injection. The wound was irrigated with saline and closed with wound clips. After survival (Control 32-33d; Experimental 40-61d), the animals were deeply anesthetized with a Euthanasia solution (IP; 40 mg/kg) and perfused through the heart using a peristaltic pump first with PBS with 0.25% procaine, and then with a fixative of 4% paraformaldehyde in 0.1 M phosphate buffer (PB; pH 7.3). The brains were removed, soaked in the fixative containing 20% sucrose, and then subsequently cut (40 µm) on a freezing microtome and sections collected in PB. Injections in all cases were verified in the SN.

### Immunohistochemistry

A 1∶4 series of sections from injected brains were rinsed three times in stock serum (PB containing 0.3% triton and serum from the secondary antibody), and then soaked in the stock serum solution containing either mouse anti-tyrosine hydroxylase (TH; 1∶7500; ImmunoStar, Inc.) or α-synuclein (1∶20,000) overnight on a shaker table at room temperature. A 1∶8 series was processed similarly for rabbit anti-glial fibrillary acidic protein (GFAP; 1∶3500; Abcam, Inc.) or mouse monoclonal anti-CD11b/c (OX-42; 1∶8000; Abcam, Inc.). The next morning the tissues were washed three times in PB, and again in stock serum, and then soaked for one hour in stock serum to which the secondary antibody had been added. Secondary antibodies were rat-adsorbed biotinylated donkey anti-mouse (1∶500; Sigma) or biotinylated goat anti-rabbit (1∶500; Sigma). After washing 3 more times, the sections were incubated in Vectastain ABC Elite solution (1∶200; Vector Laboratories) for 1 hour, washed in three rinses of PB, and reacted with diaminobenzidine dihydrochloride (DAB) intensified with nickel ammonium sulfate for 4–10 min. The sections then were counterstained with Neutral Red, dehydrated in an ascending series of alcohols, defatted in xylenes, and cover slipped with Permount. Photomicrographs were taken with a digital camera and saved with Northern Eclipse software (Empix Imaging, Inc.). Images were processed in Adobe Photoshop software (version 7.0), adjusting them with levels, brightness and contrast.

### Densitometry

The density of the striatum was calculated (Northern Eclipse) both ipsilateral and contralateral to the DOPAL injections at five different rostrocaudal levels, creating an N = 30 for either side. Sections surveyed were separated by approximately 200 microns; care was made to avoid the bundles of fibers in the globus pallidus, which was not sampled. Since the striatum sampled included both the black immunohistochemical precipitate as well as the neurons stained with Neutral Red, densitometry measurements also were obtained from 20 sections of the striatum which were processed similarly (with antibodies against CGRP and vasopressin) but had no immunohistochemical precipitate in their striata. All sections showed a narrow range of scores, with an average density of 60.4. This number was considered baseline background, and was subtracted from the densities with immunopreciptate ipsilateral and contralateral to the DOPAL injections so that the loss of immunoreactivity could be calculated.

### Unbiased Stereology

Unbiased stereological methods [36], [40], [55] were used to analyze total volume of the SN as well as the neuronal loss in its *pars compacta* (SNpc) and *pars reticulata* (SNpr) subnuclei after the DOPAL injections. SNpc and SNpr subnuclei were determined by anatomical landmarks and regional variations in cell density, orientation and morphology as outlined by others [40], [55]. The boundaries of the SNpc and SNpr were outlined in the sections with an E800 Nikon microscope through a 10× objective equipped with a motorized stage and a stereological imaging system (StereoInvestigator; MicroBrightField, Inc.). The SN subnuclei were reconstructed serially and their volume calculated with StereoInvestigator software.

Unbiased stereology was performed with the 100× objective of the microscope. The optical fractionator stereological probe was used to determine neuronal loss in the SNpc and SNpr in sections stained for both Nissl and α-synuclein and dopaminergic neuron loss in another series of sections immunostained for TH after DOPAL injections. Sampling grid sizes were 140 µm ×140 µm (area, 19600 µm^2^) with an unbiased counting frame (25×25 µ, 625 µm^2^). A guard height of 2 µm was used on sections between 10.5–13.0 µm thick. Only cells coming into focus through the sampling brick were counted. Eight to 10 sections were analyzed/case; 102–125 sampling sites for control cases and 85–126 sampling sites after DOPAL injections were analyzed in the SNpc while 252–320 sampling sites were analyzed for control cases and 212–314 sampling sites in the SNpr after DOPAL injections (the larger SNpr required more sampling sites).

### Data Analysis

Means and standard errors (M ± S.E.) were determined for experimental and control groups. Group differences for rotational asymmetry, neuronal counts and densitometry were determined using T-tests (SPSS software; v. 13). The number of neurons stained with Nissl in the SNpc and SNpr of injected brains were compared to control brains while TH-stained neurons were compared between injected and non-injected sides. The changes in behavior of the rats after injections of DOPAL were compared to the percentage loss of DA neurons.

The Paired Samples T-test was used to calculate significance for densitometry measurements, a similar test by difference method for counts of TH neurons, while the Independent Samples T-test was used for rotational asymmetry and counts of Nissl-stained neurons. Data are presented as M ± S.E. and p-values considered less than p<0.05 were considered significant. Graphs were drawn with GraphPad Prism software. Box plots present a vertical view of the data and show the shape of its distribution, its central value, and its spread. The box itself represents 50% of the data, 75^th^ percentile marks the top of the box, the 25^th^ percentile marks the bottom, while the median (50^th^ percentile) is shown as a line through the box. Whiskers show the most extreme (maximum and minimum) values in the data set and extend a maximum of 1.5 times the range in the box. Data outside these parameters are considered outliers. Outliers for the present study (not illustrated) were associated only with a single neuronal count (4817) of Nissl-stained neurons ipsilateral to the DOPAL injections.
