# Correction: The Neurotoxicity of DOPAL: Behavioral and Stereological Evidence for Its Role in Parkinson Disease Pathogenesis

**DOI:** 10.1371/annotation/819cbf4a-7e3e-4e4e-a449-b667bc6e8957

**Published:** 2011-02-26

**Authors:** W. Michael Panneton, V. B. Kumar, Qi Gan, William J. Burke, James E. Galvin

An author affiliation is missing. The second author, V. B. Kumar, is also affiliated with the Division of Geriatric Research, Education and Clinical Center, VA Medical Center, St. Louis, Missouri, United States of America.

